# Broken Umbilical Vein Catheter as an Embolus in a Neonate- An Unusual Preventable Complication

**Published:** 2013-10-01

**Authors:** Anjan Kumar Dhua, Bijender Singh, Dilip Kumar, Neeraj Awasthy

**Affiliations:** 1 Consultant Pediatric Surgeon, Pushpanjali Crosslay Hospital, Vaishali, Ghaziabad; 2 Consultant Neonatologist, Pushpanjali Crosslay Hospital, Vaishali, Ghaziabad; 3 Consultant Pediatric Cardiologist, Fortis Escorts Heart Institute, New Delhi

**Keywords:** Umbilical vein catheter, Catheter fracture, Catheter embolus, Endovascular retrieval

## Abstract

Umbilical vein catheter (UVC) is used in managing critically sick neonates all over the world. It is generally considered to be safe although various complications can arise and are well known. Herein we describe a successful retrieval of a broken and migrated UVC across the heart in a neonate. Pertinent literature regarding rarity of its occurrence and mechanism of occurrence has been touched upon to prevent such untoward complications.

## INTRODUCTION

Umbilical vein catheters (UVC) are universally used in critically sick neonates in intensive care units and they may be life-saving. When in vein they are used both for intravenous fluids and for administering medications [1]. When used properly, they are generally safe but there are various complications associated with the UVC. Herein we describe a neonate with a broken UVC lodged in the right atrium and the ends in superior Vena cava (SVC) and Inferior vena cava (IVC) and its successful uneventful removal. A search of the literature reveals that this type of complication pertaining to UVC is rare and only a handful of cases have been described in the English literature as isolated case reports.

## CASE REPORT

A 34-week male neonate weighing 1.8 kg was admitted in NICU for meconium aspiration syndrome. A 3.5 Fr UVC was used during initial days of his stay. Once the neonate was stable and UVC was no longer required it was planned for removal. On 4th day of his life removal was attempted but during catheter removal the UVC got divided by a scalpel at the skin level while removing the retaining suture. An attempt to retrieve the fractured portion of UVC was planned by a local exploration but the end had retracted into the lumen of the umbilical vein and it was not visible. A skiagram [Fig-1] showed the lower end of the UVC to lie near the base of the umbilicus.

**Figure:**
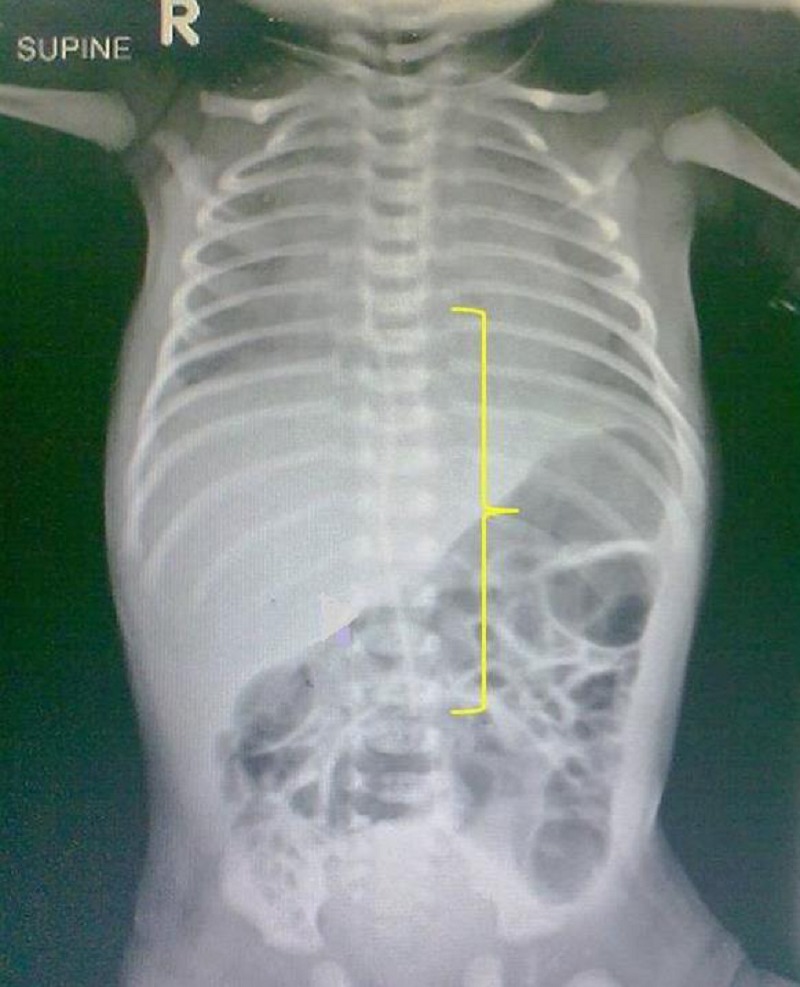
Figure 1: Fractured segment of UVC in midline (along the parenthesis) with upper end just above diaphragm and lower end at level of L3 vertebral body.

An exploration by a supra umbilical transverse incision was attempted. This was performed in NICU with full relaxation after endotracheal intubation. This decision was to ensure that no time is lost in retrieving the fragment. Despite our efforts we did not find the UVC within the umbilical vein, hence the incision was closed. A repeat skiagram done soon after showed the UVC within the right atrium with the upper end in SVC and lower end in IVC [Fig-2]. Under general anesthesia trans-femoral access was obtained by a 5 Fr sheath. A 15 mm Gooseneck snare was used through a 5 Fr multipurpose A2 catheter. The multipurpose catheter acted as a support preventing the SVC end to slip into the right atrium. The UVC was held just proximal to the tip and snared from the superior vena cava and retrieved. The patient did not have any further complications and was discharged uneventfully as per NICU protocols.

**Figure:**
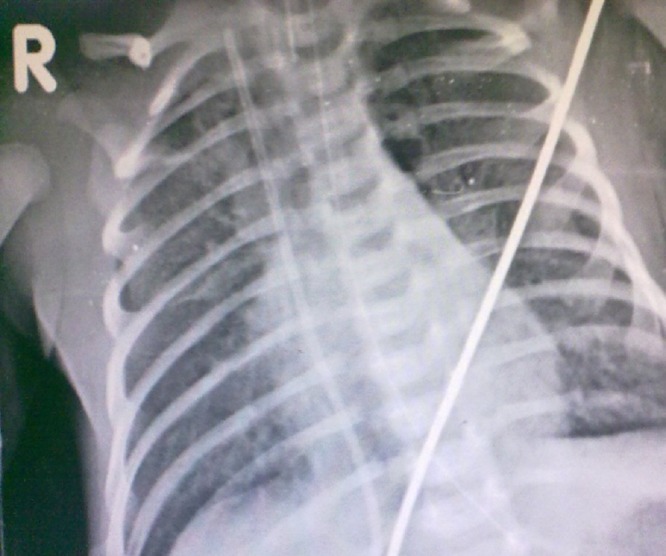
Figure 2: Migrated UVC with upper end supposedly in SVC and lower end in IVC and passing across right atrium.

## DISCUSSION

The UVC have contributed a great deal in managing critically ill patients in NICU. The arterial line is preferred for invasive monitoring while the vein is used as an access for administration of medications and intravenous fluids. These are used for the shortest time period till one has an alternative peripheral access especially in the light of various complications that have been described in the literature. These complications include nosocomial sepsis, vasospasm, vascular perforation [2], thrombosis, and emboli (air, thrombus) [3] to name a few.

With the widespread use of central venous catheters for long term parenteral nutrition, prolonged antibiotic infusion, pain therapy, chemotherapy or for hemodialysis in the adult population complications like catheter fracture with embolization have been increasingly reported [4, 5]. This phenomenon however, has been very uncommonly reported with the UVC in the newborn period. An exhaustive search of the English literature revealed only 13 articles with less than 20 cases of broken UVC in neonates [Table 1]. In a very similar case to ours Lackey et al [6] described a 3-day old neonate in whom the umbilical catheter broke during catheter insertion in the umbilical artery and migrated to the thoracic aorta. This was subsequently retrieved by open exploration. 

**Figure:**
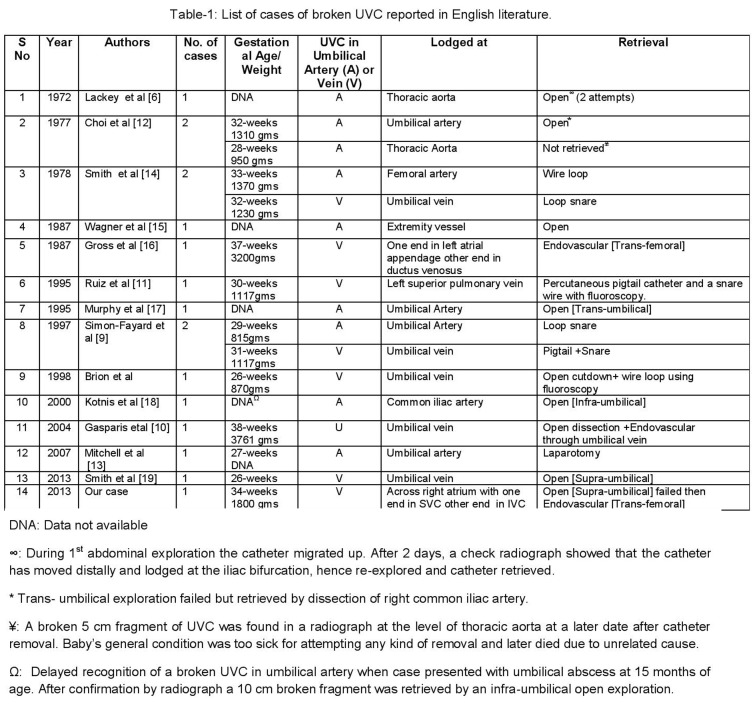
Table 1

Retrieval by endoluminal route has been used quite often in adults to retrieve various kinds of foreign body from the vascular system [7]. Similar reports with UVC in neonates have been reported only in few case reports [Table 1] [8, 9]. Gasparis et al [10] described a successful removal of a dislodged UVC through the umbilical vein using endovascular Amplatz loop snare. This minimally invasive route was also used by Ruiz et al [11] who have reported successful retrieval of a broken umbilical vein catheter lodged in the left superior pulmonary vein from a 30-week preterm neonate weighing just 1117 grams. We initially resorted to umbilical vein cut down and exploration as the lower end appeared quite close to the umbilical stump on radiography, but this attempt failed. A check radiograph revealed that the broken fragment of UVC had actually migrated higher up and hence its removal was possible only by endovascular method and it was then successfully performed by a trans-femoral approach by the pediatric interventional cardiologist using a goose-neck snare. It is a possibility that our attempt at exploration without any control on the umbilical vein may have actually pushed the fragment to migrate cranially possibly also aided by the centripetal blood flow. Endovascular retrieval techniques have an advantage in that they can be used when the ends are inaccessible and they provide a more controlled method of extraction aided by fluoroscopic guidance.

The mechanism of UVC breakage has been proposed and discussed by Choi et al [12]. He reported two cases of broken UVC and proposed that it is possible that the UVC can get inadvertently damaged by needles or scissors during catheter insertion and fixation. Subsequent attempts of removal of this weakened catheter may cause breakage. He also suggested that overzealous tightening of a purse string type suture used to secure a catheter can also weaken the wall of UVC. In our case, the catheter was divided by a surgical blade while attempting suture removal. This underscores the importance of using fine suture removal scissors especially in an active neonate who may be difficult to restrain. This would prevent similar complications and also decrease chances of personnel and patient injuries. Mitchell et al [13] while describing their experience with a broken UVC in the umbilical artery had lucidly described various steps to avoid this type of complication. As a standard practice one should always inspect the tip of the removed catheter for checking that its intactness and also insist for a check radiograph, since small broken fragment tip from these long catheters can be overlooked and missed.

Our case is the first case reported from this part of the world wherein a broken and migrated UVC was retrieved successfully. This report is intended to highlight that even a trivial procedure of UVC removal must be taken seriously and done carefully with appropriate instrumentation to prevent this kind of a rare situation. For retrieval of this type of vascular foreign body, open technique can be used when one end is accessible, else endovascular retrieval techniques should be opted as they have an edge over open technique.

## Footnotes

**Source of Support:** Nil

**Conflict of Interest:** None

